# Concentration-to-dose ratio as a predictor of lacosamide efficacy in Chinese children with epilepsy: a single-center retrospective cohort study

**DOI:** 10.3389/fphar.2026.1789142

**Published:** 2026-08-17

**Authors:** Zhaosong Du, Ying Wang, Gang Nie, Maochang Liu, Ying Li, Jun Wang

**Affiliations:** 1 Department of Pharmacy, Wuhan Children’s Hospital (Wuhan Maternal and Child Healthcare Hospital), Tongji Medical College, Huazhong University of Science and Technology, Wuhan, Hubei, China; 2 Division of Gastroenterology, Union Hospital, Tongji Medical College, Huazhong University of Science and Technology, Wuhan, Hubei, China

**Keywords:** children, concentration-to-dose ratio, efficacy, epilepsy, lacosamide, ridge regression

## Abstract

**Background and objectives:**

Lacosamide (LCM) is a third-generation concomitant antiseizure medication (ASM) that is widely used in epilepsy treatment. Reported data on the predictors of efficacy of LCM in children with epilepsy are scarce. This study aimed to explore the predictors of LCM efficacy in pediatric patients with epilepsy.

**Methods:**

This single-center retrospective cohort study was conducted in China, consecutively enrolled pediatric epilepsy patients receiving lacosamide treatment; the study period for data collection was September 2021 to April 2025. Demographic data, LCM daily dose, serum concentration, concentration-to-dose ratio (CDR), ASMs, and seizure frequency before and after treatment were collected. Patients were categorized into effective (≥50% seizure reduction) and ineffective groups. Univariate analysis and ridge regression were used to assess the impact of the factors on the efficacy of LCM. The receiver operating characteristic (ROC) curve was used to evaluate the predictive performance of the ridge regression model.

**Results:**

A total of 71 children with epilepsy were enrolled in this study (boys: 35, 49.3%; girls: 36, 50.7%). The overall effectiveness rate was 81.69%. Univariate analysis showed a significantly higher CDR in the effective group compared to the ineffective group (0.768 vs. 0.58, P = 0.031). In this exploratory pilot analysis, ridge regression implied that CDR has a positive effect on the efficacy of LCM. The AUC of the predictive performance of the ridge regression model was 0.767 (95% CI: 0.651–0.875).

**Discussion:**

CDR, reflecting weight- and dose-adjusted LCM exposure, is a predictor of LCM efficacy in pediatric epilepsy. These findings support the use of CDR for guiding individualized LCM therapy. Further multicenter studies with larger samples are warranted to validate these results and establish an optimal CDR range.

## 1 Introduction

Affecting 0.5%–1% children, epilepsy is one of the most common disorders requiring daily treatment with ASMs from childhood to adulthood (Menon and Helen Cross, 2025). Reducing the harm caused by epilepsy to patients and the burden on society makes early drug intervention particularly crucial. LCM is a third-generation antiseizure medication officially approved in China for monotherapy treatment of patients aged 4 years and above with focal seizures in 2021. Due to its superior pharmacological properties, many international guidelines for diagnosing and treating epilepsy have included it as an adjunctive treatment for generalized epilepsy and as a first-line recommendation for focal epilepsy (Jones et al., 2023; Kanner et al., 2018).

In adults, lacosamide appears to represent a reasonable alternative to levetiracetam and valproate for control of status epilepticus and has similar efficacy and safety with other third-generation antiseizure medications for the treatment of focal-onset seizures (Hahn et al., 2025; Lattanzi et al., 2022). However, due to the differences in pharmacokinetics between children and adults, the treatment of drugs becomes even more complicated in the child population. On the one hand, increased LCM clearance in children can decrease concentration, and LCM serum concentration too low can significantly increase the risk of treatment failure (Johannessen Landmark et al., 2020; Ahn et al., 2022). On the other hand, clinical practice may lead to adverse reactions by adjusting the dosage to increase LCM concentration (Fong et al., 2023; Biton et al., 2015). Given the characteristics of LCM serum concentration-dependent adverse reactions, this issue has become a critical factor limiting the rational use of drugs in clinical practice. Management of epilepsy in child patients requires balancing the risk of harm from failed therapy and the risk of adverse reactions from increased LCM exposure due to unnecessary dose increases. However, there is still no consensus on the therapeutic concentration range for it, and the studies on the efficacy of LCM in treating children with epilepsy in Chinese are scarce.

The purpose of this research was to construct a predictive model for the therapeutic effect after the use of LCM in Chinese children with epilepsy using real-world data from a single-center retrospective cohort study.

## 2 Methods and materials

### 2.1 Study design

This retrospective cohort study was conducted according to the STROBE (Strengthening the Reporting of Observational studies in Epidemiology) statement (von et al., 2007) and TRIPOD (Transparent Reporting of a multivariable prediction model for Individual Prognosis or Diagnosis) statement (Collins et al., 2015; Peduzzi et al., 1996). The data of children in this research were collected from the electronic medical record system of Wuhan Children’s Hospital from September 2021 to April 2025. The protocol was assessed and approved by the Medical Ethics Committee of Wuhan Children’s Hospital. The data was first accessed for research purposes on 03/09/2025, and the collection was completed on 16/10/2025. Due to the nature of this retrospective study, the requirement for informed consent has been waived. Ethics Batch Number: 2025R033-E01.

### 2.2 Participants

Children who were diagnosed with “epilepsy” and who take LCM in the outpatient and inpatient care were analyzed. The following demographic information was collected: age, sex, weight, LCM daily dose, LCM serum concentration, combined with other antiseizure medications, liver function, and renal function. The seizure frequency of child patients for 3 months before and after the use of LCM was determined from the electronic medical record system. The inclusion criteria are: (1) the first time use of LCM after diagnosis of epilepsy; (2) carrying out LCM serum concentration monitoring; (3) regular use of LCM for at least 1 month. The exclusion criteria are: (1) with any underlying diseases; (2) who have not undergone serum concentration monitoring of LCM; (3) with missing information; (4) with abnormal liver and renal functions.

### 2.3 The dosage regimen of lacosamide and blood sampling timing

All child patients were administered the oral formulation of lacosamide. The initial dose was 2 mg·kg^-1^ per day, administered in two doses. The maintenance dose was gradually increased weekly, with each time the daily dosage being increased by 2 mg·kg^-1^. The dose was adjusted according to the efficacy and tolerability to reach the target dose of 4–12 mg·kg^-1^·d^-1^, with the maximum dose being 400 mg·d^-1^.

Blood sampling for steady-state drug concentration measurement was performed after stabilizing the medication dosage following treatment initiation. The interval between stabilizing the medication dosage and sampling timing needs to exceed five elimination half-lives of lacosamide (approximately 13 h), ensuring that steady-state conditions were achieved for all patients prior to sampling. The venous blood samples of each patient were collected on an empty stomach the next morning.

### 2.4 Definitions

The frequency of seizure episodes within 1 month prior to the use of lacosamide was set as the baseline seizure frequency. The frequency of epilepsy decreased by more than 50% within 1 month after the use of lacosamide was categorized as effective. If the frequency of epilepsy was less than once within 1 month prior to the use of lacosamide, then compare the frequency within 3 months before and after the medication. Simultaneously, the child patients were divided into an effective group and an ineffective group according to their frequency of seizures after LCM treatment.

Due to significant inter-individual variations, the serum concentration of LCM at steady-state was adjusted based on each patient’s body weight and dosage. This concentration was expressed as the concentration-to-dose ratio of LCM (CDR, (μg·ml^-1^)/(mg·kg^-1^)). The CDR was calculated by dividing the LCM serum concentration by the adjusted daily dose of LCM.

### 2.5 Statistical analysis

Data analyses were performed using R 4.5.1 software. We used the graphical method and the Shapiro-Wilk method to test the normality of the data. For continuous variables that did not conform to the normal distribution, we described the data as the median (interquartile range) [*M* (P25, P75)]. Child patients’ information was compared between the effective group and the ineffective group. Statistical significance was calculated with the Wilcoxon Rank Sum Test. For categorical variables, we described the data as percentages (%), and statistical significance between two groups was calculated with the Chi-square test or Fisher’s exact test (when any expected cell count was <5). Given potential correlations among CDR, concentration, and dose, we employed ridge regression to handle multicollinearity (Hoerl and Kennard, 2000). This approach penalizes the model complexity and yields stable coefficient estimates without excluding clinically relevant variables. We employed ridge regression analysis using the glmnet package (alpha = 0) to investigate the association of LCM serum concentration with efficacy, aiming to identify the most closely related predictive factors to the LCM efficacy from a large number of candidate factors, thereby constructing a clinically practical risk assessment tool. Due to the limited sample size and particularly the small number of ineffective cases (n = 13), the study is underpowered for definitive predictive modelling. Consequently, all analyses are considered exploratory. We used ridge regression to handle multicollinearity, but no causal or confirmatory inferences should be drawn from the results. The reported coefficients and thresholds should be interpreted as pilot data for future hypothesis-testing studies.

The covariates considered were: weight, age, sex, daily dose, LCM serum concentration, CDR, and drug combination. We modelled weight, age, daily dose, LCM serum concentration, and CDR as continuous variables. We categorized the drug combination as the LCM monotherapy group and the LCM combination with other antiseizure medications (ASMs) group. We used the receiver operating characteristic (ROC) curve to evaluate the predictive performance of the ridge regression model. The linearity assumption in the logit for the continuous variable was evaluated using the Box-Tidwell test. Multicollinearity was evaluated by using the Pearson correlation coefficient and examining the Variance Inflation Factor in a multiple regression model with identical dependent and independent variables. To assess the stability and potential overfitting of the ridge regression model, we performed bootstrap internal validation with 1,000 resamples (Steyerberg et al., 2001). Calibration was examined using the bootstrap-corrected predicted probabilities; observed proportions and 95% binomial confidence intervals were plotted against the mean predicted probabilities. We used the Hosmer-Lemeshow goodness-of-fit Chi-square test to assess the fit of the model. All statistical hypothesis tests were two-sided and conducted at a significance level of 0.05.

## 3 Results

### 3.1 Patient demographic and clinical characteristics

A total of 71 pediatric patients with epilepsy were included in this study. The demographic and clinical characteristics of the patients are summarized in Table 1. The median age of the children was 7.2 years (IQR: 5.485–9.36), with the majority (80.28%) belonging to the 4–12 years age group. The sex distribution was nearly equal (35 boys, 49.3%; 36 girls, 50.7%). The median weight was 26 kg (IQR: 20–33.75). The median LCM daily dose was 4 mg·kg^-1^ (IQR: 3.33–5), with a corresponding median serum concentration of 2.68 μg·ml^-1^ (IQR: 2.22–3.545). The median concentration-to-dose ratio (CDR) was 0.76 (IQR: 0.573–0.924). Liver and renal function parameters, including ALT, AST, ALP, CR, UA, and BUN, were all within normal ranges. The most commonly concomitant antiseizure medications (ASMs) were valproate (VPA, 21.13%) and levetiracetam (LEV, 18.31%). The overall effectiveness rate of LCM therapy was 81.69%.

**TABLE 1:** Demographics and clinical characteristics of the children with epilepsy.

Characteristics	Value
Age [*M* (P25, P75), year]	7.2 (5.485,9.36)
Age group [n (%)]	
≤4 years	7 (9.86)
4–12 years	57 (80.28)
>12years	7 (9.86)
Sex [n (%)]	
Boy	35 (49.3)
Girl	36 (50.7)
Weight [*M* (P25, P75), kg]	26 (20,33.75)
Daily dose [*M* (P25, P75), mg·kg^-1^]	4 (3.33,5)
LCM serum concentration [*M* (P25, P75), μg·ml^-1^]	2.68 (2.22,3.545)
CDR [*M* (P25, P75), (μg·ml^-1^)/(mg·kg^-1^)]	0.76 (0.573,0.924)
ALT [*M* (P25, P75), U·L^-1^]	12 (10,15.5)
AST [*M* (P25, P75), U·L^-1^]	24 (20,28.5)
ALP [*M* (P25, P75), U·L^-1^]	243 (205,291)
CR [*M* (P25, P75), μmol·L^-1^]	36.6 (31.65,41.9)
UA [*M* (P25, P75), μmol·L^-1^]	258 (236,346)
BUN [*M* (P25, P75), mmol·L^-1^]	4.2 (3.55,4.8)
Concomitant ASMs [n (%)]	
VPA	15 (21.13)
LEV	13 (18.31)
OXC	8 (11.27)
PMP	3 (4.23)
Others	6 (8.45)
Effect [n (%)]	
Effective	58 (81.69)
Ineffective	13 (18.31)

*LCM*: lacosamide; *CDR*: concentration-to-dose ratio; *ALT*: alanine transaminase; *AST*: aspartate transaminase; *ALP*: alkaline phosphatase; *CR*: creatinine; *UA*: uric acid; *BUN*: blood urea nitrogen; *ASMs*: antiseizure medications; *VPA*: valproic acid; *LEV*: levetiracetam; *OXC*: oxcarbazepine; *PMP*: perampanel.

### 3.2 Univariate analysis of factors affecting LCM efficacy

The patients were divided into an effective group (n = 58) and an ineffective group (n = 13) for comparative analysis, as shown in Table 2. The results indicated no statistically significant differences between the two groups in terms of age, sex, weight, daily dose, LCM serum concentration, or most biochemical parameters (P > 0.05). However, the CDR was significantly higher in the effective group compared to the ineffective group (0.768 vs. 0.58, P = 0.031). Although the difference in sex distribution did not reach statistical significance (P = 0.063), it suggested a potential trend towards better efficacy in boys.

**TABLE 2:** Influence factors affecting the efficacy of LCM therapy in child patients with epilepsy.

Variable	Effective group (n = 58)	Ineffective group (n = 13)	W/*χ* ^2^	*P* value
Age [*M* (P25, P75), year]	7.8 (5.54,9.65)	6.63 (5.46,7.18)	287.5	0.186
Sex (boy/girl)	32/26	3/10	—	0.063†
Weight [*M* (P25, P75), kg]	26 (20,34.9)	23 (20,26)	277	0.139
Daily dose [*M* (P25, P75), mg·kg^-1^]	3.92 (3.33,4.88)	4.35 (3.85,5)	457.5	0.234
LCM serum concentration [*M* (P25, P75), μg·ml^-1^]	2.8 (2.24,3.62)	2.3 (2.2,3.11)	283.5	0.167
CDR [*M* (P25, P75), (μg·ml^-1^)/(mg·kg^-1^)]	0.768 (0.592,0.94)	0.58 (0.469,0.804)	231	0.031
ALT [*M* (P25, P75), U·L^-1^]	11 (10,15)	12 (11,18)	452.5	0.263
AST [*M* (P25, P75), U·L^-1^]	24 (20,27.8)	25 (19,31)	418	0.546
ALP [*M* (P25, P75), U·L^-1^]	251 (208,290)	205 (189,299)	284.5	0.171
CR [*M* (P25, P75), μmol·L^-1^]	38 (32.5,43.4)	32.8 (27.2,38.4)	247	0.054
UA [*M* (P25, P75), μmol·L^-1^]	260 (235,358)	253 (240,287)	308.5	0.312
BUN [*M* (P25, P75), mmol·L^-1^]	4.2 (3.52,4.8)	4 (3.7,5.2)	359.5	0.800
Concomitant ASMs [n (%)]				
LCM monotherapy	31 (43.66)	8 (11.27)	0.0491	0.825
LCM + ASMs	27 (38.03)	5 (7.04)	​	​

Baseline comparisons were exploratory; *P*-values are presented without adjustment for multiple testing and should be interpreted with caution. †Fisher’s exact test applied for sex comparison (male-ineffective cell n = 3, expected count <5), producing P = 0.063. *LCM*: lacosamide; *CDR*: concentration-to-dose ratio; *ALT*: alanine transaminase; *AST*: aspartate transaminase; *ALP*: alkaline phosphatase; *CR*: creatinine; *UA*: uric acid; *BUN*: blood urea nitrogen; *ASMs*: antiseizure medications; *VPA*: valproic acid; *LEV*: levetiracetam; *OXC*: oxcarbazepine; *PMP*: perampanel.

### 3.3 Ridge regression analysis of predictors of efficacy of LCM

**FIGURE 1:**
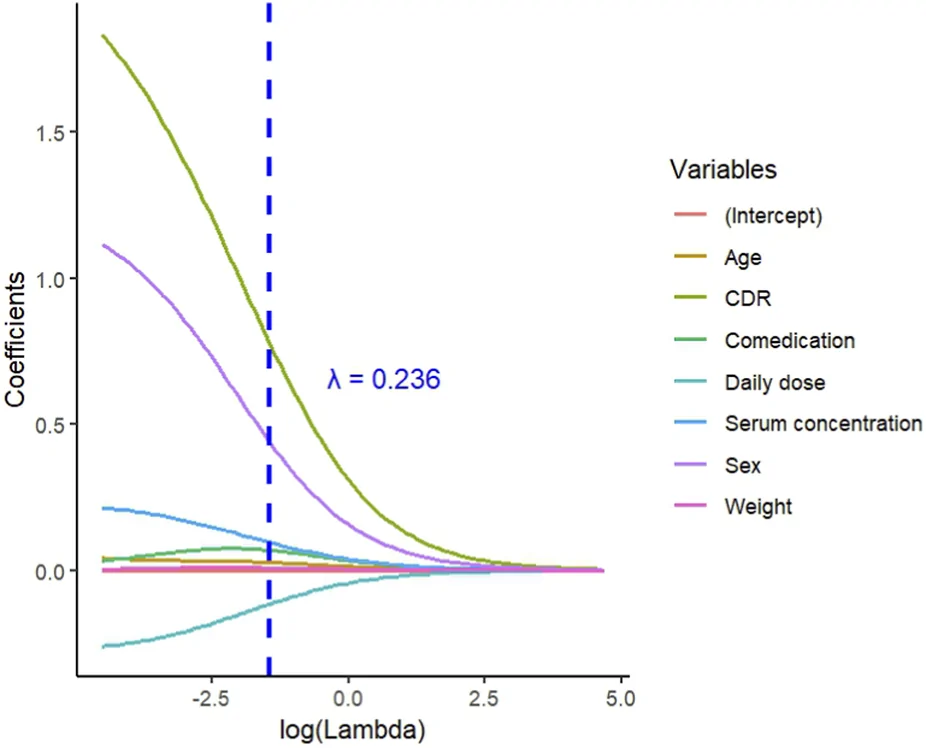
Ridge trace diagram. Each colored line represents a variable. The blue dotted line indicates the position of the optimal value.

The VIF values of the variables and the results of the Box-Tidwell test are presented in the Supplementary Table. The VIF values for the three variables (CDR, serum concentration, and daily dose) were 18.992, 19.850, and 12.810, respectively (Supplementary Table 1). To further identify predictive factors of the efficacy of LCM, a ridge regression analysis was performed (The optimal regularization parameter was determined to be 0.236 through 10-fold cross-validation. λ = 0.236. Figure 1). The results are presented in Table 3. CDR was identified as an important predictive factor that has a positive effect on the efficacy of LCM. Daily dose, serum concentration, concomitant ASMs, age, and weight have a relatively small impact on the efficacy of LCM.

**TABLE 3:** Ridge regression analyses of the predictors of LCM efficacy.

Variable	Coefficients
Intercept	0.479
CDR	0.776
Sex	
Girl (reference)	​
Boy	0.439
Daily dose	−0.116
Serum concentration	0.095
Comedication	
LCM monotherapy (reference)	
LCM + ASMs	0.068
Age	0.027
Weight	0.008

*LCM*: lacosamide; *CDR*: concentration-to-dose ratio; *ASMs*: antiseizure medications.

### 3.4 Model prediction performance

**FIGURE 2:**
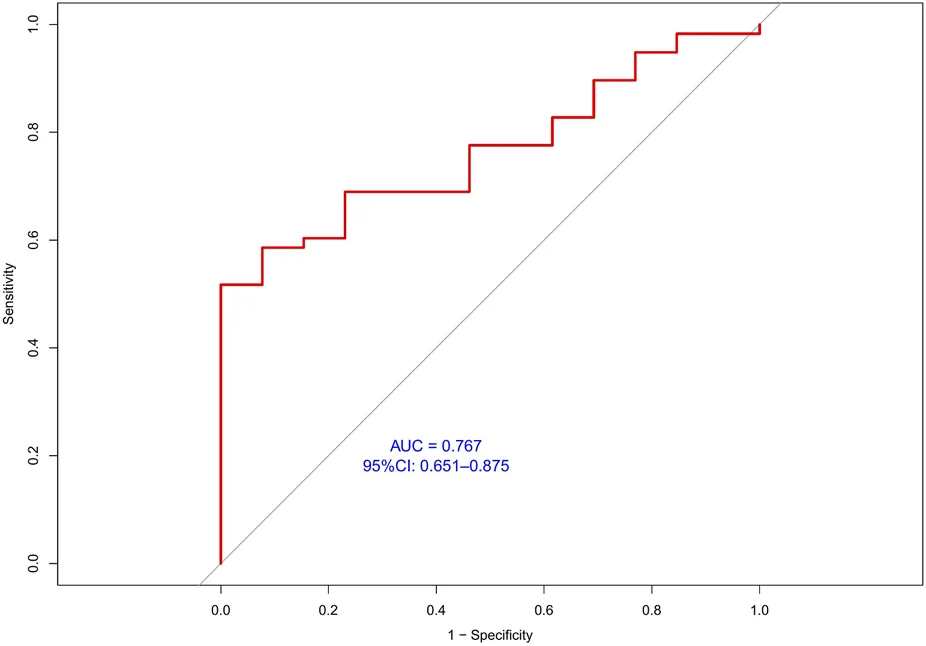
Ridge regression model ROC curve.

The predictive performance of the ridge regression model was evaluated using the ROC curve (Figure 2). The results showed that the AUC of the model was 0.767 (95% CI: 0.651–0.875), indicating the model has an acceptable predictive value for the efficacy of LCM in pediatric patients with epilepsy.

### 3.5 Validation of the efficacy prediction model for LCM

**FIGURE 3:**
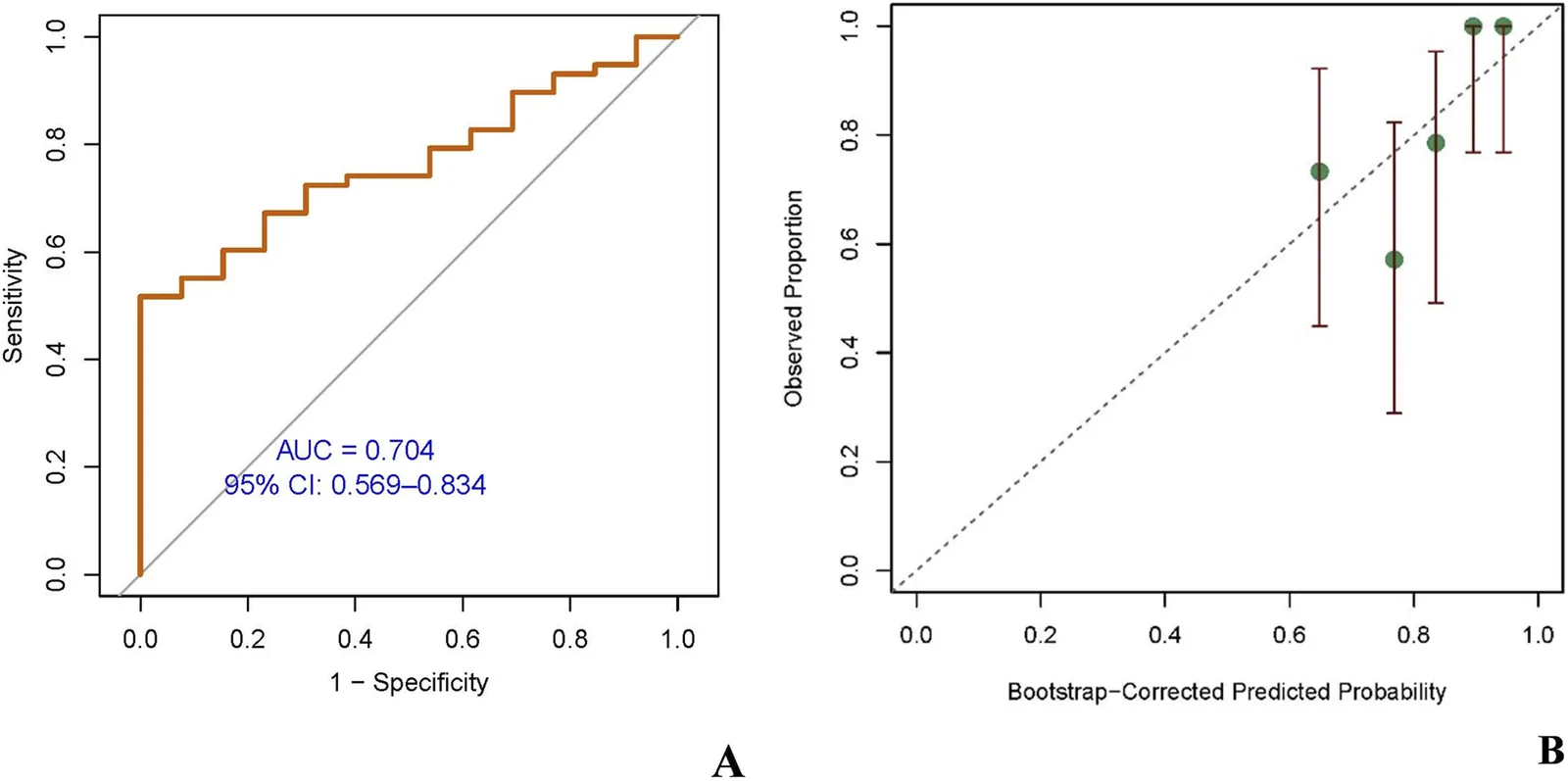
Internal validation curve and calibration curve of the LCM efficacy prediction model. **(A)** Internal validation ROC curve; **(B)** Calibration curve.

The bootstrap repeated subsampling method was used to validate the prediction model. The ROC curve was drawn to evaluate the performance of the model (Figure 3A). The AUC of the internal validation group was 0.704 (95% CI: 0.569–0.834). Calibration of the prediction model was performed using calibration curves and the Hosmer-Lemeshow goodness-of-fit test. Calibration was assessed graphically in Figure 3B. Five groups were used due to the small sample size (n = 71), consistent with recommendations for samples under 100. The Hosmer-Lemeshow test was not significant (χ^2^ (3) = 6.371, g = 5, P = 0.095); however, given the limited sample size and the exploratory nature of this analysis, external validation in independent cohorts is warranted to confirm these findings.

### 3.6 Clinically actionable thresholds analysis

The optimal CDR threshold was 0.61, yielding a sensitivity of 0.74 and a specificity of 0.62. Due to the small sample size of the ineffective group (n = 13), this threshold and performance indicators are only exploratory and need to be verified in a larger sample.

### 3.7 Intergroup comparison of serum concentration and CDR

**FIGURE 4:**
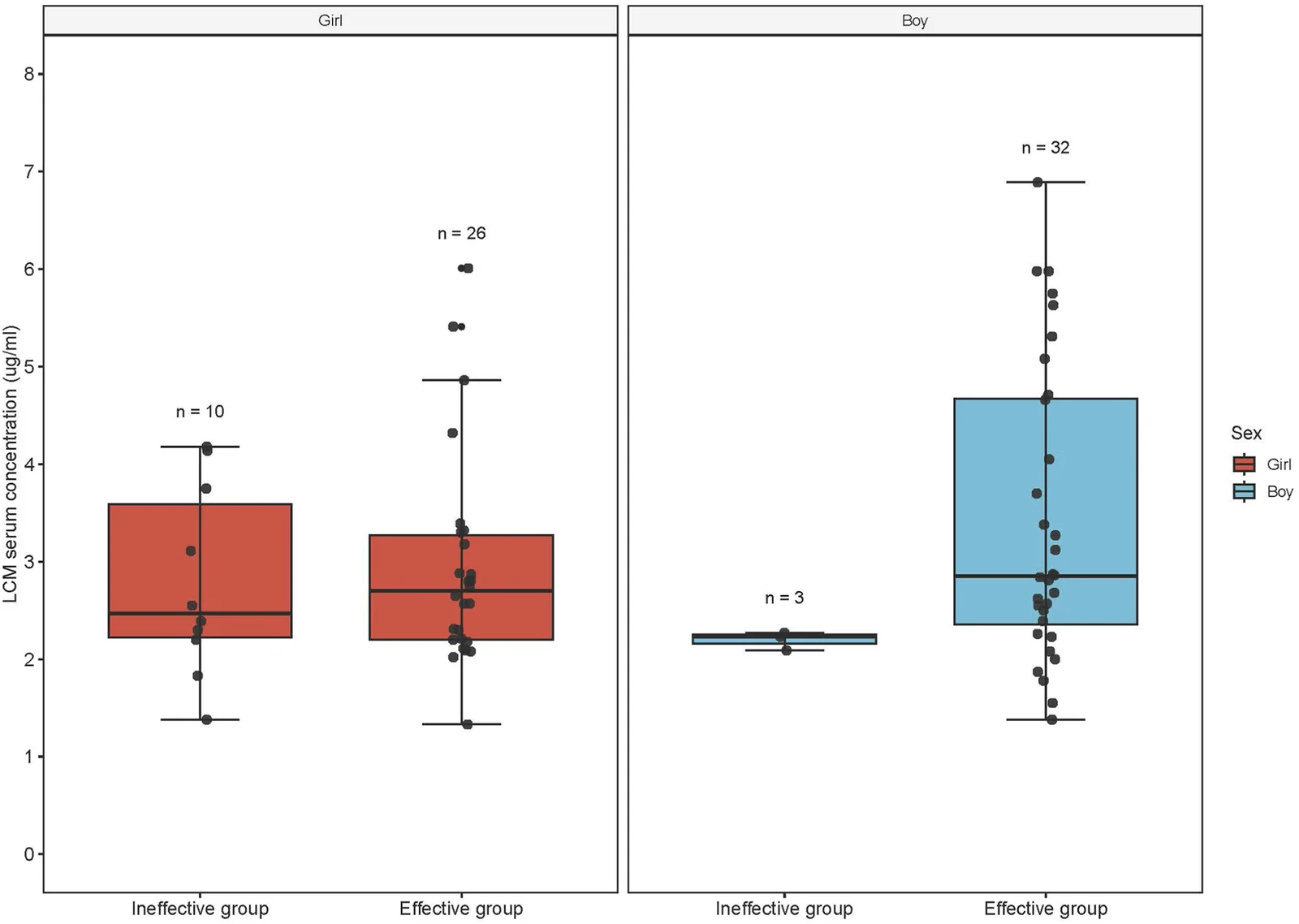
Compare the LCM serum concentration between the ineffective group and the effective group. The differences in LCM serum concentration between the effective group and the ineffective group were compared separately for both boys and girls. Statistics performed by the Wilcoxon Rank Sum Test.

**FIGURE 5:**
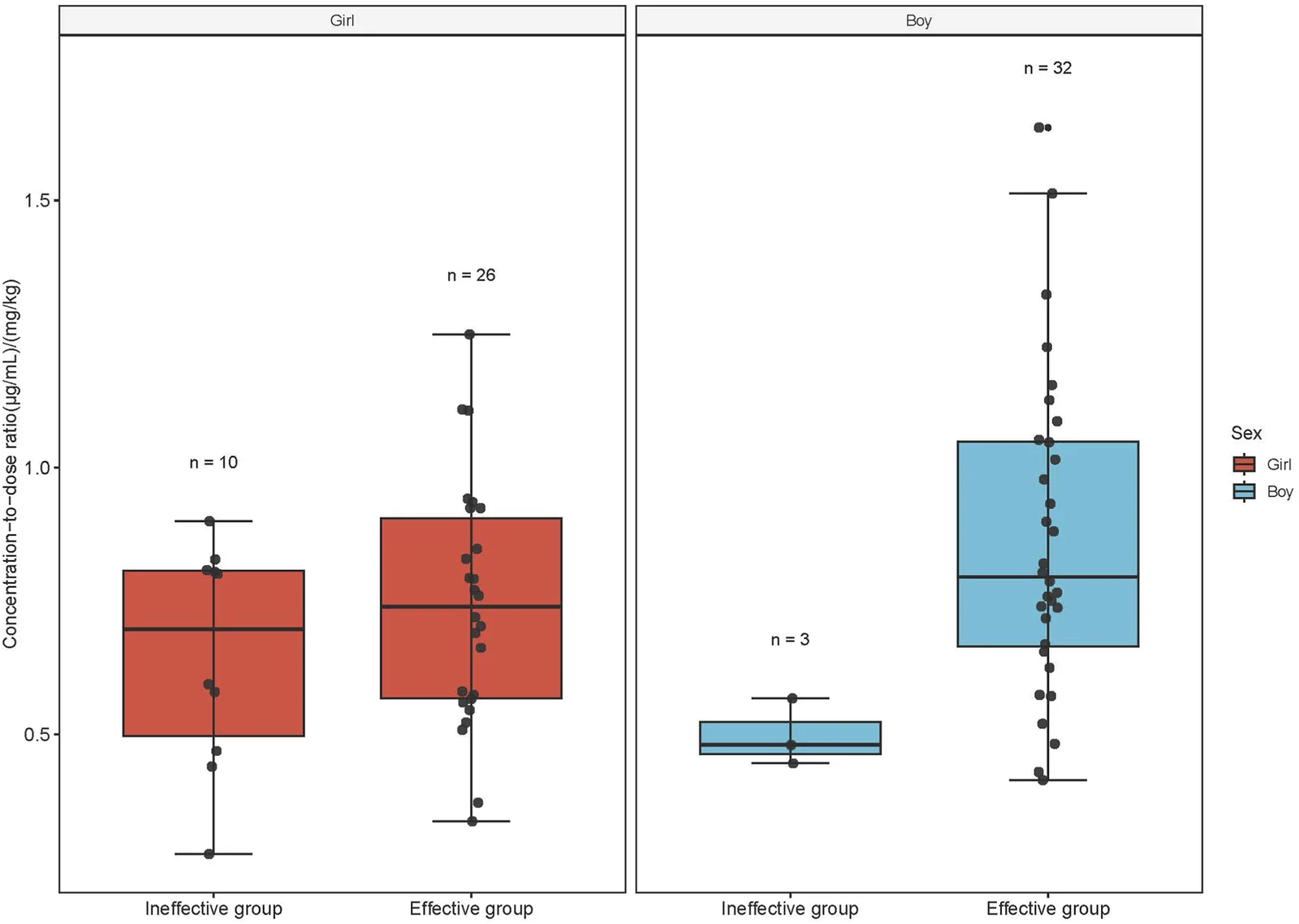
Compare the CDR between the ineffective group and the effective group. The differences in CDR between the effective group and the ineffective group were compared separately for both boys and girls. Statistics performed by the Wilcoxon Rank Sum Test. When comparing the effective group and the ineffective group among the boys, *P* = 0.013 (n = 3 in the ineffective group among the boys; these results should be interpreted with caution).

In a further subgroup analysis, the pediatric patients with epilepsy were divided into the boy and the girl groups for comparison. Figures 4, 5 illustrate the comparisons of LCM serum concentration and CDR, respectively, between different sex groups in the effective and ineffective groups. In the girl subgroup, the LCM serum concentration and the CDR were slightly lower in the ineffective group, but the difference was not statistically significant. In contrast, in the boy subgroup, although there was no significant difference in the LCM serum concentration, the CDR was higher in the effective group, further supporting its potential value in predicting treatment outcome. It is worth noting that, due to the strict inclusion criteria, only 3 boys in the ineffective group may be susceptible to bias in the results of this study.

## 4 Discussions

There are few published studies regarding the influence factors of LCM efficacy in Chinese pediatric patients with epilepsy. In this exploratory pilot study, we evaluated the differences among different variables between the effective group and the ineffective group through a retrospective analysis. Although the daily dose of LCM and the LCM serum concentration were similar between the effective group and the ineffective group, the CDR was significantly higher in the effective group. With our ridge regression model, we identified the CDR in pediatric patients with epilepsy that can be used by clinicians to individualize drug administration more effectively.

In general, the exposure level of ASMs was believed to significantly affect the efficacy (Simon and von Fabeck, 2025). Meanwhile, most antiepileptic drugs also have recommended therapeutic concentration ranges (Johannessen Landmark et al., 2020; Patsalos et al., 2008). However, the reference range for LCM is not well established, with effective concentration levels ranging from 2.2 to 20 mg/L (Schultz and Mahmoud, 2020; Kohn et al., 2023). Furthermore, studies on the influence of LCM serum concentration on therapeutic efficacy are scarce and inconsistent. According to the report, LCM at any dose was found to be over one-and-a-half times more likely to reduce seizures than placebo (Babar et al., 2021). A study investigating whether the clinical efficacy of newer ASMs was associated with plasma levels of these ASMs showed that plasma levels of LCM at the time of a single blood draw were not associated with seizure control (Hentschel et al., 2024). On the contrary, a prospective study included 215 pediatric patients with epilepsy who were under LCM showed that LCM plasma concentrations were strongly associated with its clinical efficacy and toxicity (Zhao et al., 2024). Based on the contradictory research results mentioned above, it is highly necessary to conduct an in-depth study on the relationship between LCM serum concentration and its efficacy. In this study, data show that the efficacy of LCM was not correlated with its serum concentration. After the LCM serum concentration had been corrected by dosage, the CDR was higher in the effective group than in the ineffective group. Our results highlighted that measuring serum concentrations of LCM can play a valuable role in guiding patient management. The efficacy cannot be fully evaluated merely based on the original drug concentration, as the dosage differences mask crucial information. By combining dosage and body weight correction, it is possible to more accurately identify the pharmacokinetic differences among patients and guide individualized drug administration.

The biotransformation of LCM mainly proceeds via dual pathways: renal elimination of the unchanged drug and metabolic degradation of the drug by cytochrome P450 (CYP) enzymes. CYP-catalyzed oxidation is the major metabolic pathway for LCM in adults, mediated by CYP450, mainly CYP2C19, accounting for about 60% of the dose. Approximately 40% of the dose is excreted in the urine as the unchanged active drug (Cawello, 2015). The *CYP2C19* gene has a high degree of polymorphism, and the wild-type CYP2C19*1 is a functional allele mediated by CYP2C19 metabolism. CYP2C19*2 and CYP2C19*3 are the main alleles that cause functional defects of the CYP2C19 enzyme and can reduce the activity of the CYP2C19 enzyme. CYP2C19*17 is a function-enhancing allele that can enhance the activity of the CYP2C19 enzyme. The population differences of *CYP2C19* gene polymorphism are significant among different ethnic groups. In the Chinese population, the high frequencies of alleles were *CYP2C19∗2* (30.36%), with the most common diplotype being *CYP2C19* ∗1/∗2 (37.62%) (Luan et al., 2026). In contrast to the Chinese population, a previous study found that the increased activity allele CYP2C19*17 was most prevalent in Central Europe (25%–33%) (Petrović et al., 2019). The distribution characteristics of this genetic variation have ethnic specificity and are one of the important genetic bases that affect drug metabolic phenotypes and individualized clinical medication.

In children, a previous study indicated that the main difference between sexes is the UGT enzyme system, while the difference in the CYP enzyme system was relatively small (Verdez et al., 2022). Thus, LCM has a relatively similar metabolism in different sexes, which explains the result of univariate analysis in the study, namely that there is no difference in the efficacy of LCM between different sexes. Regarding the impact of sex on the efficacy of LCM, a few more reports have also reached similar conclusions. A real-world study included 105 children (1 month–4 years) with focal seizures treated with adjunctive lacosamide therapy, suggesting that there was no difference in gender between the effective group and the ineffective group (Yang et al., 2024). In our previous study, we confirmed that the exposure level of LCM has no significant difference between the boy group and the girl group (Du et al., 2025). These consistent results showed that the efficacy of LCM is not affected by sex. However, the results of ridge regression in this study indicated that sex has a positive effect on the efficacy of LCM. In the subgroup analysis of the boys’ group, we found the CDR was higher in the effective group compared to the ineffective group. This research is inconsistent with the results of previous studies, which may be explained by the following assumption: The boy has a relatively low proportion of fat in his body, which will affect the distribution volume. In adults, a clinical study involving 565 individuals with epilepsy showed an effect on Cmax at steady state of approximately 8% attributed to body fat (Carona et al., 2021; Schaefer et al., 2015). In children, research has shown that there are significant differences in body fat distribution between boys and girls, especially after 11 years old (Pang et al., 2024; Weber et al., 2013). However, the influence of body fat distribution on the efficacy of LCM has not been reported yet in children. Further in-depth research will still be necessary in the future.

LCM is often used as an adjunctive medication for the treatment of children with epilepsy (Wu et al., 2025). In view of the fact that children with epilepsy face a higher risk of various neurologic (e.g., cognitive impairment) and psychological (e.g., mood disorders, attention-deficit/hyperactivity disorder) disorders, some of which may be associated with the use of AEDs (Wei and Lee, 2015), exploring the influence of concomitant ASMs on the efficacy of LCM has important significance for the rational clinical use. Previous studies have shown that various ASMs can affect the pharmacokinetics of LCM in child patients with epilepsy (Mao et al., 2025; Woods et al., 2024; Lukka et al., 2021). However, the combined use of ASMs results in different outcomes in terms of the efficacy of LCM. A double-blind, randomized, parallel-group trial involving 255 patients aged 1month to 4years with uncontrolled focal seizures showed adjunctive LCM did not show superior efficacy versus placebo (Makedonska et al., 2024). In another double-blind, randomized, placebo-controlled trial, researchers evaluated the efficacy of adjunctive LCM in children and adolescents aged 4–17years with uncontrolled focal seizures. The results indicated LCM was efficacious regardless of whether sodium channel-blocking (SCB) AEDs were part of the concomitant treatment regimen (Farkas et al., 2019). In line with previous studies, we found there was no difference in whether to combine antiseizure medications between the effective group and the ineffective group.

### 4.1 Limitations

Similar to other studies, this research inevitably has certain limitations. Firstly, this was a single-center study, which might limit the external validity of our findings. Secondly, the sample size of this study is relatively small due to the strict inclusion criteria, especially only 13 patients were included in the ineffective group, and only 3 of them were boys, which may weaken the credibility of the results. Meanwhile, the subgroup analysis by sex is exploratory. No formal interaction test was conducted, so differences between boys and girls should be interpreted cautiously. Thirdly, this study did not investigate the influence of the *CYP2C19* gene polymorphism on the efficacy of LCM. Fourthly, the evaluation period for the baseline seizure frequency, which inconsistently varies between 1 month and 3 months, may also bias the evaluation of drug efficacy. However, a previous study has shown that among epilepsy patients, the response rate of 50% has a similar weight in clinical decision-making at both 1-month and 3-month outcomes (Yang et al., 2023). Hence, in this study of children with epilepsy, the 1-month status can serve as an effective substitute for the 3-month status. Finally, relevant confounders were not collected in the data (e.g., type of epilepsy, disease duration, epilepsy etiology, medication adherence), highlighting a more comprehensive study is needed. It should be noted that because CDR is derived from dose and concentration, its observed association with efficacy should be interpreted with caution, and our results merely indicate a correlation between CDR and LCM efficacy, but do not establish a causal link. Regression to the mean may partially explain the observed seizure reduction, as patients were enrolled during high-frequency periods. This statistical phenomenon could lead to an overestimation of treatment efficacy, especially given the lack of a concurrent control group. Future studies with longer baseline periods and sham-controlled designs are warranted to disentangle true drug effects from natural fluctuations. Nevertheless, there are few studies on the predictive factors of LCM’s efficacy among children. Our conclusion still holds significant importance for the rational clinical use of LCM in the treatment of epilepsy.

## 5 Conclusion

In conclusion, we found that CDR is a potential predictive factor of the efficacy of LCM in pediatric patients with epilepsy. The serum concentration of LCM after dose-weight correction can provide a reference for individualized medication in clinical practice. Future studies with large sample sizes and multiple centers will be needed to determine the optimal range of LCM CDR in child patients with epilepsy.

## Data Availability

The raw data supporting the conclusions of this article will be made available by the authors, without undue reservation.
