# Supplementary material for: Concentration-to-dose ratio as a predictor of lacosamide efficacy in Chinese children with epilepsy: a single-center retrospective cohort study
Source: Front Pharmacol. 2026 Aug 17;17:1789142. doi: 10.3389/fphar.2026.1789142 (PMC13526605; doi:10.3389/fphar.2026.1789142)
Supplement: Supplementary file 1 [file Supplementaryfile1.docx]

| Variable | VIF value |
| --- | --- |
| Age | 4.492 |
| Sex | 1.131 |
| Weight | 4.798 |
| Daily dose | 12.81 |
| Concentration | 19.85 |
| CDR | 18.992 |
| Comedication | 1.095 |

Supplementary table 1 VIF value of the variable

*VIF:* Variance Inflation Factor; *CDR:* concentration-to-dose ratio

Supplementary table 2 the results of Box-Tidwell test for the continuous variable

| Variable | Statistic | P value |
| --- | --- | --- |
| Age | 0.335 | 0.739 |
| Weight | -0.728 | 0.469 |
| Daily dose | 0.481 | 0.632 |
| Concentration | -0.526 | 0.601 |
| CDR | 0.681 | 0.498 |

*CDR:* concentration-to-dose ratio
